# Keratoconus prevalence in astigmatic adolescents: findings from a nationwide screening setting

**DOI:** 10.1038/s41433-025-03995-9

**Published:** 2025-09-18

**Authors:** Margarita Safir, Itay Nitzan, Yair Hanina, Dan Heller, Michael Mimouni, Nir Sorkin

**Affiliations:** 1 Department of Ophthalmology, Rabin Medical Center, Faculty of Medicine, Tel Aviv University, Tel Aviv, Israel; 2 Department of Military Medicine, Faculty of Medicine, Hebrew University of Jerusalem, Jerusalem, Israel; 3 Department of Ophthalmology, Hadassah-Hebrew University Medical Center, Jerusalem, Israel; 4 Department of Ophthalmology, Rambam Health Care Campus, Bruce and Ruth Rappaport Faculty of Medicine, Technion-Israel Institute of Technology, Haifa, Israel; 5 Department of Ophthalmology, Tel Aviv Medical Center and Sackler Faculty of Medicine, Tel Aviv University, Tel Aviv, Israel

**Keywords:** Risk factors, Corneal diseases

## Abstract

**Objectives:**

To assess the association between varying levels of astigmatism and the likelihood of keratoconus diagnosis in adolescents.

**Methods:**

This cross-sectional study included 896,377 adolescents aged 16–20 years who underwent a standardised medical assessment between 2011 and 2022, including refraction and topography/tomography in cases where astigmatism above 2.00 dioptres (D) was observed. Astigmatism was categorised into five groups: None, 0.75− < 2.00 D, 2.00– < 3.00 D, 3.00– < 5.00 D, and ≥5.00 D. Logistic regression models were used to assess the association between astigmatism and keratoconus diagnosis. Receiver Operating Characteristic (ROC) curve analysis was performed to evaluate the discriminatory ability of cylinder power in detecting keratoconus.

**Results:**

Overall, 1886 adolescents (0.21%) were diagnosed with keratoconus. Increasing astigmatism levels were strongly associated with higher keratoconus prevalence, rising from 0.1% for 0.75– < 2.00 D to 17.4% for ≥5.00 D (*p* < 0.001). Each 1-diopter increase in cylinder power above 2.00 D was linked to a 1.76-fold increase in keratoconus odds (OR = 1.76, 95% CI: 1.70–1.82, *p* < 0.001). Astigmatism axis demonstrated limited discriminatory ability. ROC analysis showed moderate discriminatory power for cylinder power (AUC = 0.752), with a cut-off of 2.88 D yielding a sensitivity of 0.744 and a specificity of 0.644.

**Conclusions:**

In this large cohort of adolescents, increasing astigmatism power was significantly associated with keratoconus diagnosis. These findings suggest that combining astigmatism thresholds with other clinical factors may enhance screening strategies, enabling timely intervention to prevent disease progression.

## Introduction

Keratoconus is a progressive corneal disorder characterised by thinning and cone-shaped protrusion of the cornea, leading to increasing magnitude of irregular astigmatism and visual impairment [1]. Most keratoconus cases are diagnosed during puberty and early twenties [1, 2]. Early diagnosis is crucial, as untreated keratoconus can result in significant visual disability and reduction of quality of life [3–6]. While corneal topography and tomography have long been established as the gold standard for diagnosing keratoconus [1, 7, 8], these tools are not routinely available in most ophthalmology clinics and are typically reserved for patients with a moderate to high suspicion of keratoconus rather than used as screening tools. Therefore, identifying early, readily accessible indicators of keratoconus is of great clinical importance.

Astigmatism, particularly higher magnitude astigmatism, is a well-known refractive error that often accompanies keratoconus [1]. Previous studies have indicated a relationship between higher degrees of astigmatism and keratoconus [1, 9, 10], suggesting that astigmatism might serve as a clinical marker for the condition. However, this association has yet to be fully characterised in large, population-based studies.

In Israel, the majority of the 16–18 year-old population undergoes a standardised evaluation including refraction by an optometrist or ophthalmologist, followed by corneal topography or tomography to rule out keratoconus when astigmatism above 2.00 D is observed. This population-wide screening program provides a unique opportunity to explore the prevalence and risk factors associated with keratoconus in a large, representative cohort of adolescents. Thus, this study aimed to assess the relationship between different levels of astigmatism and the likelihood of keratoconus diagnosis.

## Methods

### Ethics

This study was approved by the Institutional Review Board of the IDF medical corps and adhered to the tenets of the Declaration of Helsinki.

### Patient evaluation

The Israeli military pre-conscription assessment involves a comprehensive medical evaluation and classification for fitness to serve, conducted between the ages of 16 and 18. Given the mandatory nature of military service in Israel, this screening program offers a unique opportunity for a population-wide, cross-sectional evaluation of adolescents. The assessment includes standardised questionnaires completed by the applicant and their general physician, as well as a visual acuity and refractive examination performed by an ophthalmologist or optometrist. An autorefractometer is used for all refractive assessments, and subjective refraction is performed at the discretion of the examining clinician when deemed necessary. However, the specific type of refractive assessment (i.e., subjective vs. objective) used in each individual case is not recorded and therefore could not be analysed. Adolescents with astigmatism of 2.00 D or more are routinely referred for corneal topography/tomography (various models, not available for analysis) and a follow-up ophthalmologist consultation to rule out corneal ectasia [11]. The diagnosis of keratoconus is made at the discretion of community-based ophthalmologists, based on corneal topography or tomography findings in conjunction with the clinical history. For cases of astigmatism <2.00 D, no preconscription screening policy is defined. Thus, referral for further examination in these cases is at the discretion of community ophthalmologists or optometrists.

### Statistical analysis

Statistical analyses were performed with SPSS version 29.0 (IBM Corp., Armonk, NY, USA). The Chi-square test was used to compare categorical variables and the independent samples t-test for continuous variables, as appropriate. The prevalence of keratoconus was calculated for each cylinder power and axis group. Logistic regression models were utilised to examine the association between keratoconus and each of the following independent variables: cylinder power (analysed as both continuous and categorical), anisoastigmatism, and enantiomorphism. All models were adjusted for sex and birth year. Results are presented as odds ratios (OR) with 95% confidence intervals (CI). A two-sided *p*-value < 0.05 was considered statistically significant. A ROC (Receiver Operating Characteristic) curve was plotted for cylinder power, with the area under the curve (AUC) and Youden Index calculated. The logistic regression and ROC curve analyses included only adolescents referred for corneal topography/tomography based on astigmatism screening ( ≥ 2 D). This criterion ensured a consistent diagnostic approach for evaluating keratoconus, enabling a valid assessment of astigmatism as an indicator within this population.

For the purpose of analysis, anisoastigmatism was defined as an absolute interocular difference of ≥1.00 dioptre in cylindrical power, consistent with established clinical thresholds. Enantiomorphism was defined as mirror symmetry in astigmatic axis orientation between the eyes. To assess this, we computed the interocular axis difference after mirroring the left eye’s axis as if viewed from the right side. If the resulting axis difference was within ±15°, the pair was classified as enantiomorphic; otherwise, as non-enantiomorphic. Astigmatism orientation was categorised using standard definitions from our prior work: with-the-rule (WTR) astigmatism: steep meridian at 90° ± 30°, against-the-rule (ATR) astigmatism: steep meridian at 180° ± 30°, oblique (OBL) astigmatism: steep meridian between 31°–59° or 121°–149°.

## Results

**Fig. 1:**
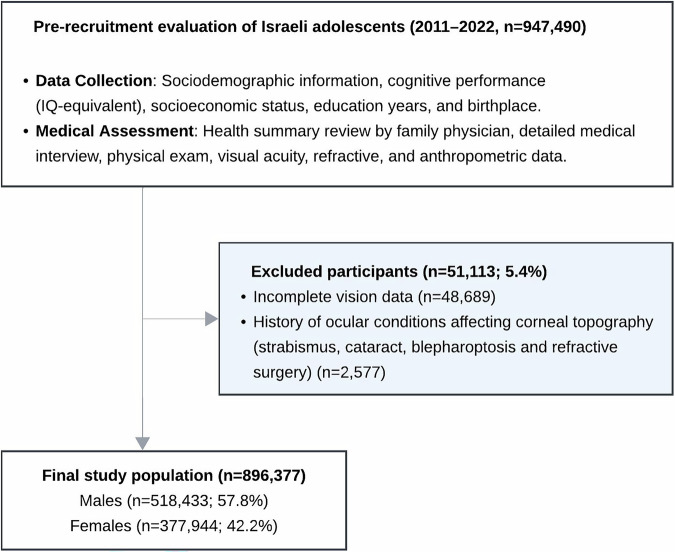
Study exclusion process. Patients were excluded from analysis if refractive or visual acuity data were missing, or in cases where other ophthalmologic conditions potentially affecting overall astigmatism coexisted.

Among the 947,490 adolescents assessed, 51,113 (5.4%) were excluded due to incomplete visual acuity or refractive data or other ophthalmologic conditions potentially affecting overall astigmatism (Fig. 1). The final sample included 896,377 adolescents, with 518,433 males (57.8%) and 377,944 females (42.2%). The mean age was 17.2 ± 0.8 years, distributed similarly between those with and without keratoconus (17.6 ± 0.9 and 17.2 ± 0.8, *p* < 0.001). The study population comprised 1886 (0.21%) adolescents with keratoconus and 894,491 without. Compared with non-keratoconus participants, those with keratoconus were more likely to be male (70.1% vs. 29.9%, *p* < 0.001).

### Astigmatism and keratoconus

Astigmatism ( > 0.75 D) was present in 164,079 (18.3%) participants. Of these, 134,963 (15.1%) had astigmatism between 0.75 D and <2.00 D, 18,132 (2.0%) had astigmatism between 2.00 D and <3.00 D, 8,863 (1.0%) had astigmatism between 3.00 D and <5.00 D, and 2,121 (0.2%) had astigmatism ≥5.00 D. The prevalence of keratoconus rose with increasing astigmatism levels, showing prevalence of 0.0% for <0.75 D, 0.1% for 0.75– < 2.00 D, 2.3% for 2.00– < 3.00 D, 9.5% for 3.00– < 5.00 D, and 17.4% for ≥5.00 D (*p* < 0.001; Table 1). Among participants with against-the-rule (ATR) astigmatism, keratoconus was identified in 357 (0.7%) individuals, compared to 1057 (1.1%) among those with with-the-rule (WTR) astigmatism, and 343 (1.6%) in those with oblique (OBL) astigmatism (*p* < 0.001; Table 2).

**Table 1:** Keratoconus prevalence by astigmatism power groups.

		Without KC	With KC	Total	*p*-value
**Astigmatism (D)**	< 0.75	732,169 (100.0)	129 (0.0)	732,298	<0.001^a^
	≥0.75, <2	134,829 (99.9)	134 (0.1)	134,963	
	≥2, <3	17,717 (97.7)	415 (2.3)	18,132	
	≥3, <5	8024 (90.5)	839 (9.5)	8863	
	≥5	1752 (82.6)	369 (17.4)	2121	

Data shown as the absolute number and row percentage of participants.

^a^*p*-value obtained from the Chi-Square test.

**Table 2:** Keratoconus prevalence by astigmatism axis groups.

		Without KC	With KC	Total	*p*-value
**Astigmatism axis**	<0.75	732,169 (100.0)	129 (0.0)	732,298	<0.001^a^
	ATR	47,634 (99.3)	357 (0.7)	47,991	
	WTR	93,818 (98.9)	1,057 (1.1)	94,875	
	OBL	20,870 (98.4)	343 (1.6)	21,213	

*ATR* against the rule; *WTR* with the rule, *OBL* oblique.

Data shown as the absolute number and row percentage of participants.

^a^*p*-value obtained from the Chi-Square test.

After excluding adolescents not referred for corneal topography/tomography based on their screening refraction, 29,116 participants remained, with 1623 (5.57%) eventually diagnosed with keratoconus. Of the 29,116 participants, 13,663 (46.9%) were anisometropic and 18,023 (61.9%) had enantiomorphism of cylinder axes. The prevalence of keratoconus was higher among adolescents with anisoastigmatism (1060/13,663; 7.8%) compared to those without anisoastigmatism (563/15,453; 3.6%). It was also higher among those without enantiomorphism (778/11,093; 7.0%) compared to those with enantiomorphism (845/18,023; 4.7%). Both differences were statistically significant (*p* < 0.001).

Logistic regression analyses showed that higher cylinder power, presence of anisoastigmatism, and absence of enantiomorphism were significantly associated with an increased likelihood of keratoconus, after adjusting for sex and birth year. When cylinder power was analysed as a continuous variable, each 1-diopter increase was associated with a 1.75-fold higher likelihood of keratoconus (OR = 1.75, 95% CI: 1.69–1.80, *p* < 0.001). When treated categorically, the likelihood of keratoconus increased with higher levels of astigmatism, reaching an OR of 4.45 (95% CI: 3.94–5.02, *p* < 0.001) for astigmatism levels of 3.00– < 5.00 D and an OR of 8.80 (95% CI: 7.59–10.22, *p* < 0.001) for levels ≥5.00 D, compared to the reference category of 2.00– < 3.00 D. Additionally, adolescents with anisoastigmatism had 2.20-fold higher likelihood of keratoconus compared to those without anisoastigmatism (OR = 2.20, 95% CI: 1.98–2.45, *p* < 0.001). The absence of enantiomorphism was also associated with an increased likelihood of keratoconus, with a 1.52 times higher likelihood compared to adolescents with enantiomorphism (OR = 1.52, 95% CI: 1.37–1.68, *p* < 0.001).

### ROC curve

**Fig. 2:**
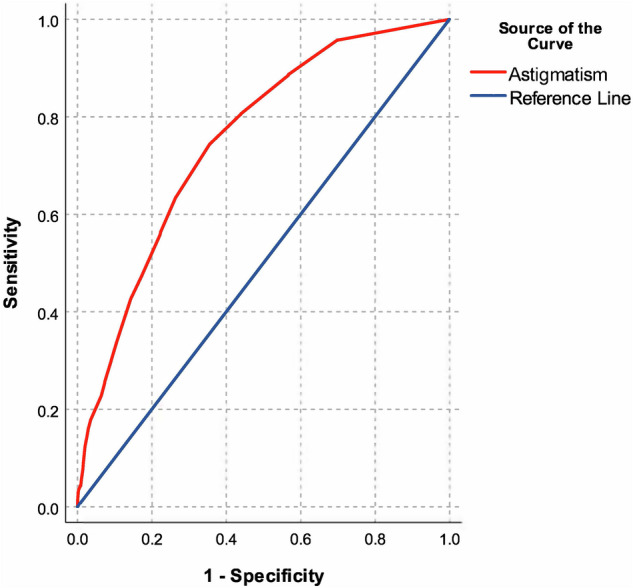
Receiver Operating Characteristic (ROC) curve analysis to evaluate the discriminatory ability of cylinder power in detecting keratoconus. Cylinder power alone demonstrated a moderate discriminatory ability for detecting keratoconus, with an area under the curve of 0.752 (95% CI: 0.740–0.763, *p* < 0.001).

A ROC curve analysis demonstrated that cylinder power alone has a moderate discriminatory ability for detecting keratoconus, with an area under the curve (AUC) of 0.752 (95% CI: 0.740–0.763, *p* < 0.001; Fig. 2). The Youden Index was maximised at a cut-off value of 2.88 D, corresponding to a sensitivity of 0.744 and a specificity of 0.644 (Table S1). When stratified by astigmatism axis groups, the AUC remained consistent among adolescents with WTR, ATR, and oblique astigmatism (Fig. S1).

## Discussion

In this large, population-based study of nearly 900,000 adolescents, we observed a significant correlation between increasing levels of astigmatism and the likelihood of keratoconus. Notably, cylinder power greater than 2.00 D was strongly associated with keratoconus, with the prevalence reaching 9.5% for astigmatism levels of 3.00– < 5.00 D and 17.4% for ≥5.00 D. This is the largest study thus far addressing this issue, and its findings are consistent with previous smaller studies that have identified high astigmatism as a hallmark feature of keratoconus progression [12], and keratoconic eyes versus healthy ones [9, 13, 14].

While the results of our ROC analysis further support the strong association between astigmatism and keratoconus, it seems that the utility of astigmatism as a potential single screening tool for keratoconus may be limited. A cylinder power cut-off value of 2.88 D yielded moderate sensitivity and specificity for detecting keratoconus. This cut-off value aligns with the findings of Kim et al. who assessed corneal tomography of 64 patients with keratoconus, yielding mean total astigmatic power of 3.05 D [13]. However, as this cut-off demonstrates moderate sensitivity and specificity, it should be used for keratoconus screening only in conjunction with other clinical factors, such as a history of eye rubbing, atopic ocular disease and a family history of keratoconus in order to enhance screening effectiveness [1, 15, 16].

Keratoconus is recognised as a bilateral asymmetric disorder [17, 18], which is supported by our findings that both anisoastigmatism and lack of enantiomorphism were found to be significantly related to keratoconus diagnosis in the current cohort. However, the odds ratios for the aforementioned findings were less pronounced than the odds for high astigmatism power. This may be explained by the fact that aniso-astigmatism ≥1.00 D often places at least one eye in the total astigmatism ≥2.00 D group, which is already a well-established marker for keratoconus. Consequently, the presence of aniso-astigmatism does not offer significant additional discriminatory power beyond what is captured by the total astigmatism threshold. Additionally, since keratoconus frequently manifests with varying degrees of astigmatism in both eyes, the overall level of astigmatism in each eye is more informative than the difference between the two eyes. These findings suggest that while anisoastigmatism can be a characteristic feature of keratoconus, it may not be as reliable for early detection compared to other parameters such as total astigmatism power or topographic indices. Astigmatism axis, while demonstrating oblique and WTR axis predominance for keratoconus in concordance with previous reports [10], was not found to be a valuable screening tool in the current study as well.

Notably, 263 individuals were diagnosed with keratoconus despite having astigmatism levels lower than 2.00 D, likely due to first degree family history of corneal ectatic disease or other clinical features raising suspicion for keratoconus (e.g., atopic conjunctival disorders and/or eye rubbing) which led to their referral for corneal topography. These additional contributing factors were unfortunately outside the scope of our dataset since the referral for examination was performed by community ophthalmologist prior to the preconscription evaluation. Therefore, this unique subgroup was excluded from further analyses, allowing us to assess more precisely the utility of our current practice screening referral threshold of 2.00 D of astigmatism.

While our study benefits from a large sample size, several limitations should be noted. First, the cross-sectional design limits our ability to establish future diagnosis of keratoconus in previously normal appearing corneas. Further longitudinal studies are needed in order to evaluate the value of astigmatism in predicting long-term risk for keratoconus diagnosis. Additionally, the repeatability of corneal measurements in keratoconus patients may be reduced due to corneal irregularities [9], which could introduce variability and potential misclassification into our findings. Moreover, we did not account for ocular surface disturbances, which could potentially affect keratometry readings and introduce measurement bias as well. Another limitation is the differential diagnostic workup across subgroups. Only adolescents with ≥2.00 D of astigmatism were routinely referred for corneal imaging. Therefore, individuals with <2.00 D astigmatism, even in the presence of additional risk factors (clinical features suggestive of keratoconus (e.g., family history, eye rubbing) did not undergo routine topography or tomography, unless previously referred by community ophthalmologists, potentially leading to underestimation of keratoconus prevalence in this subgroup. Finally, It is important to note that astigmatism values were examined in a population in their late teens, and the findings may not necessarily be extrapolated for younger children and adolescents [14, 19]. Further studies are needed to determine age-specific cut-off astigmatism values for keratoconus screening.

In conclusion, our study highlights the significant association between astigmatism and keratoconus in adolescents, suggesting that high astigmatism, particularly above 2.00 D, could serve as a valuable marker for early detection of keratoconus. Since keratoconus has a very significant impact on the patient’s quality of life and visual function, it is crucial to do our best to diagnose keratoconus as early as possible in order to prevent further progression and visual decline. While astigmatism alone has moderate diagnostic accuracy, its predictive value can be enhanced when combined with clinical history elements such as frequent eye rubbing, a family history of keratoconus, or atopic ocular conditions. Therefore, a practical screening strategy for high school children could involve identifying those with ≥2.00 D of astigmatism and assessing them for these additional risk factors. Our findings underscore the importance of comprehensive screening in adolescents with high astigmatism to facilitate early diagnosis and management of keratoconus, potentially improving long-term visual outcomes.

## Summary

### What was known before:

- Keratoconus is a progressive corneal ectasia typically diagnosed in adolescence or early adulthood, often associated with irregular astigmatism and visual impairment.
- Corneal topography and tomography are the gold standard for diagnosing keratoconus but are not routinely used as screening tools.
- Prior studies have suggested a link between high astigmatism and keratoconus, but most were small or lacked population-based data.

### What this study adds:

- This is the largest population-based study to date assessing the relationship between astigmatism and keratoconus in adolescents, involving nearly 900,000 individuals.
- The study found a strong association between increasing astigmatism levels and keratoconus prevalence, with maximal sensitivity and specificity above 2.75 dioptres.

## Supplementary information

Supplemental Material

## Data Availability

The datasets generated during and/or analysed during the current study are not publicly available due to confidentiality restrictions but are available from the corresponding author on reasonable request.
