# Supplementary material for: Keratoconus prevalence in astigmatic adolescents: findings from a nationwide screening setting
Source: Eye (Lond). 2025 Sep 18;39(16):2958–62. doi: 10.1038/s41433-025-03995-9 (PMC12583458; doi:10.1038/s41433-025-03995-9)
Supplement: Supplementary file 1 — Supplemental Material [file 41433_2025_3995_MOESM1_ESM.docx]

**Supplementary Material**

Table S1-**Coordinates of the ROC Curve and Youden Index**

Astigmatism values corresponding to the highest Youden Index are highlighted in yellow.

| Positive if Greater Than or Equal To | Sensitivity | 1 - Specificity | Specificity | Youden Index |
| --- | --- | --- | --- | --- |
| 1.0000 | 1.000 | 1.000 | 0.000 | 0.000 |
| 2.0100 | 0.957 | 0.702 | 0.298 | 0.256 |
| 2.0350 | 0.957 | 0.702 | 0.298 | 0.256 |
| 2.0750 | 0.957 | 0.701 | 0.299 | 0.256 |
| 2.1100 | 0.957 | 0.701 | 0.299 | 0.257 |
| 2.1350 | 0.957 | 0.700 | 0.300 | 0.257 |
| 2.1750 | 0.957 | 0.700 | 0.300 | 0.257 |
| 2.2100 | 0.957 | 0.700 | 0.300 | 0.258 |
| 2.2250 | 0.957 | 0.699 | 0.301 | 0.258 |
| 2.2350 | 0.957 | 0.699 | 0.301 | 0.258 |
| 2.2450 | 0.957 | 0.699 | 0.301 | 0.258 |
| 2.2550 | 0.887 | 0.568 | 0.432 | 0.319 |
| 2.2650 | 0.887 | 0.568 | 0.432 | 0.320 |
| 2.2750 | 0.886 | 0.567 | 0.433 | 0.319 |
| 2.2900 | 0.885 | 0.567 | 0.433 | 0.318 |
| 2.3250 | 0.885 | 0.567 | 0.433 | 0.318 |
| 2.3600 | 0.885 | 0.567 | 0.433 | 0.318 |
| 2.4250 | 0.885 | 0.567 | 0.433 | 0.319 |
| 2.4900 | 0.885 | 0.567 | 0.433 | 0.318 |
| 2.5100 | 0.806 | 0.439 | 0.561 | 0.367 |
| 2.5350 | 0.805 | 0.439 | 0.561 | 0.366 |
| 2.5600 | 0.805 | 0.439 | 0.561 | 0.366 |
| 2.5850 | 0.805 | 0.439 | 0.561 | 0.366 |
| 2.6100 | 0.805 | 0.439 | 0.561 | 0.366 |
| 2.6600 | 0.805 | 0.439 | 0.561 | 0.366 |
| 2.7250 | 0.805 | 0.439 | 0.561 | 0.366 |
| 2.7750 | 0.744 | 0.356 | 0.644 | 0.388 |
| 2.8350 | 0.744 | 0.356 | 0.644 | 0.388 |
| 2.8800 | 0.744 | 0.356 | 0.644 | 0.389 |
| 2.9200 | 0.744 | 0.356 | 0.644 | 0.389 |
| 2.9700 | 0.744 | 0.356 | 0.644 | 0.389 |
| 2.9950 | 0.744 | 0.356 | 0.644 | 0.389 |
| 3.0050 | 0.635 | 0.265 | 0.735 | 0.371 |
| 3.0200 | 0.635 | 0.265 | 0.735 | 0.371 |
| 3.0400 | 0.635 | 0.265 | 0.735 | 0.371 |
| 3.0600 | 0.635 | 0.265 | 0.735 | 0.371 |
| 3.0950 | 0.635 | 0.264 | 0.736 | 0.371 |
| 3.1600 | 0.635 | 0.264 | 0.736 | 0.371 |
| 3.2250 | 0.635 | 0.264 | 0.736 | 0.371 |
| 3.2750 | 0.563 | 0.223 | 0.777 | 0.340 |
| 3.3100 | 0.562 | 0.223 | 0.777 | 0.339 |
| 3.3350 | 0.561 | 0.223 | 0.777 | 0.338 |
| 3.3600 | 0.560 | 0.222 | 0.778 | 0.338 |
| 3.3750 | 0.559 | 0.222 | 0.778 | 0.337 |
| 3.3900 | 0.559 | 0.222 | 0.778 | 0.337 |
| 3.4500 | 0.558 | 0.222 | 0.778 | 0.336 |
| 3.5050 | 0.473 | 0.172 | 0.828 | 0.301 |
| 3.5550 | 0.473 | 0.172 | 0.828 | 0.301 |
| 3.6100 | 0.473 | 0.172 | 0.828 | 0.300 |
| 3.6600 | 0.473 | 0.172 | 0.828 | 0.300 |
| 3.7250 | 0.473 | 0.172 | 0.828 | 0.300 |
| 3.7750 | 0.426 | 0.143 | 0.857 | 0.283 |
| 3.8100 | 0.426 | 0.143 | 0.857 | 0.283 |
| 3.8350 | 0.426 | 0.143 | 0.857 | 0.283 |
| 3.9250 | 0.426 | 0.143 | 0.857 | 0.283 |
| 4.0600 | 0.338 | 0.105 | 0.895 | 0.232 |
| 4.1850 | 0.338 | 0.105 | 0.895 | 0.232 |
| 4.3100 | 0.299 | 0.090 | 0.910 | 0.209 |
| 4.3950 | 0.299 | 0.090 | 0.910 | 0.209 |
| 4.4350 | 0.299 | 0.090 | 0.910 | 0.209 |
| 4.4600 | 0.298 | 0.090 | 0.910 | 0.208 |
| 4.4850 | 0.298 | 0.090 | 0.910 | 0.208 |
| 4.5500 | 0.256 | 0.073 | 0.927 | 0.183 |
| 4.6100 | 0.256 | 0.073 | 0.927 | 0.183 |
| 4.6600 | 0.256 | 0.073 | 0.927 | 0.182 |
| 4.7250 | 0.254 | 0.073 | 0.927 | 0.181 |
| 4.8750 | 0.227 | 0.064 | 0.936 | 0.164 |
| 5.0500 | 0.178 | 0.035 | 0.965 | 0.143 |
| 5.1100 | 0.178 | 0.035 | 0.965 | 0.143 |
| 5.1600 | 0.178 | 0.035 | 0.965 | 0.143 |
| 5.2250 | 0.178 | 0.035 | 0.965 | 0.143 |
| 5.3250 | 0.163 | 0.030 | 0.970 | 0.133 |
| 5.4500 | 0.163 | 0.030 | 0.970 | 0.133 |
| 5.5100 | 0.139 | 0.024 | 0.976 | 0.115 |
| 5.5350 | 0.139 | 0.024 | 0.976 | 0.115 |
| 5.5600 | 0.139 | 0.024 | 0.976 | 0.115 |
| 5.6600 | 0.139 | 0.024 | 0.976 | 0.115 |
| 5.8750 | 0.123 | 0.020 | 0.980 | 0.103 |
| 6.1250 | 0.092 | 0.016 | 0.984 | 0.076 |
| 6.3750 | 0.077 | 0.015 | 0.985 | 0.063 |
| 6.5600 | 0.066 | 0.012 | 0.988 | 0.054 |
| 6.6250 | 0.065 | 0.012 | 0.988 | 0.053 |
| 6.6350 | 0.065 | 0.012 | 0.988 | 0.053 |
| 6.6450 | 0.065 | 0.012 | 0.988 | 0.053 |
| 6.7000 | 0.065 | 0.012 | 0.988 | 0.052 |
| 6.7750 | 0.057 | 0.011 | 0.989 | 0.046 |
| 6.9000 | 0.057 | 0.011 | 0.989 | 0.046 |
| 7.0250 | 0.043 | 0.009 | 0.991 | 0.035 |
| 7.0600 | 0.043 | 0.007 | 0.993 | 0.036 |
| 7.1600 | 0.043 | 0.007 | 0.993 | 0.036 |
| 7.3100 | 0.042 | 0.007 | 0.993 | 0.035 |
| 7.3850 | 0.041 | 0.007 | 0.993 | 0.034 |
| 7.4250 | 0.041 | 0.007 | 0.993 | 0.034 |
| 7.4750 | 0.041 | 0.007 | 0.993 | 0.035 |
| 7.6150 | 0.034 | 0.003 | 0.997 | 0.031 |
| 7.7400 | 0.034 | 0.003 | 0.997 | 0.031 |
| 7.8650 | 0.030 | 0.002 | 0.998 | 0.028 |
| 7.9900 | 0.030 | 0.002 | 0.998 | 0.028 |
| 8.1250 | 0.024 | 0.002 | 0.998 | 0.022 |
| 8.3750 | 0.023 | 0.001 | 0.999 | 0.021 |
| 8.6250 | 0.020 | 0.001 | 0.999 | 0.019 |
| 8.7750 | 0.017 | 0.001 | 0.999 | 0.016 |
| 8.8100 | 0.017 | 0.001 | 0.999 | 0.016 |
| 8.8450 | 0.017 | 0.001 | 0.999 | 0.016 |
| 8.9350 | 0.017 | 0.001 | 0.999 | 0.016 |
| 9.1250 | 0.007 | 0.000 | 1.000 | 0.007 |
| 9.3750 | 0.007 | 0.000 | 1.000 | 0.006 |
| 9.6250 | 0.004 | 0.000 | 1.000 | 0.004 |
| 9.8700 | 0.001 | 0.000 | 1.000 | 0.001 |
| 10.9900 | 0.000 | 0.000 | 1.000 | 0.000 |

**
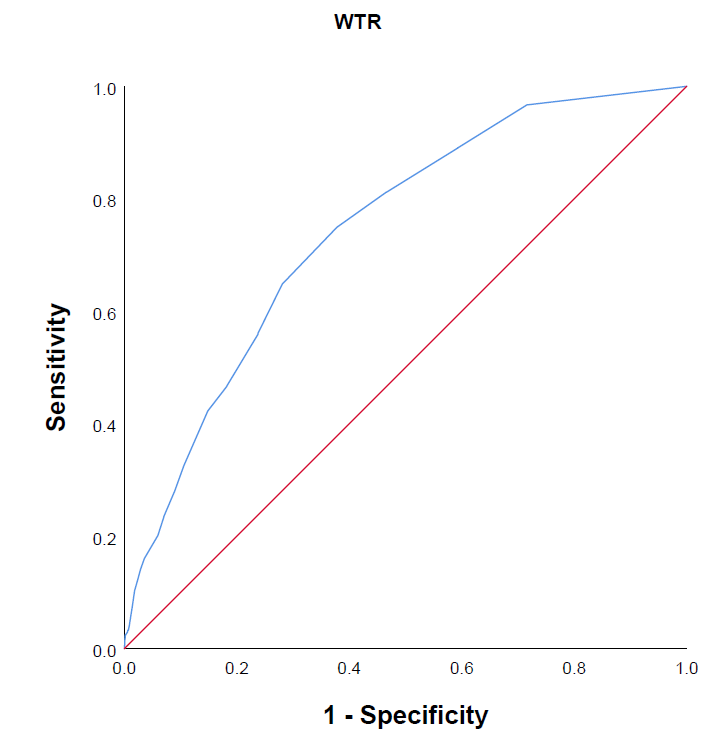

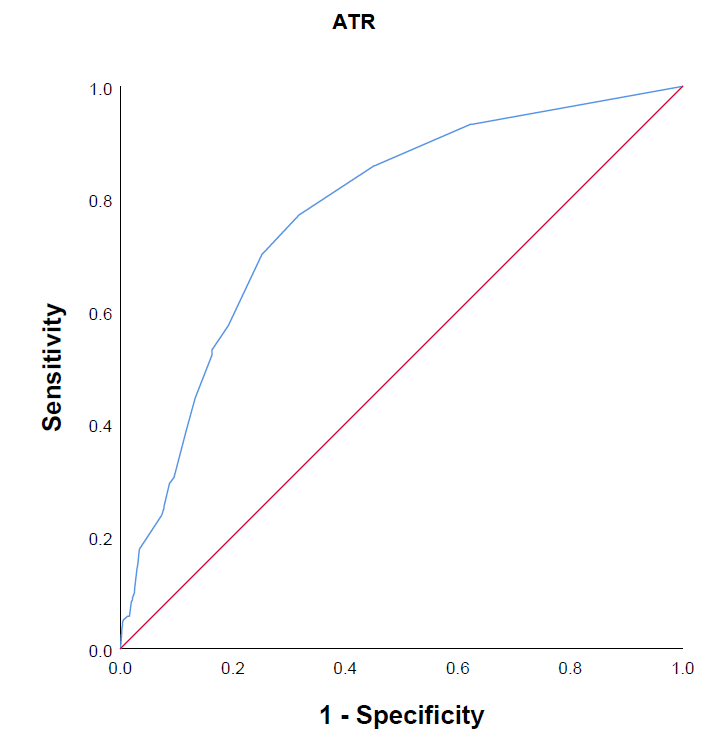

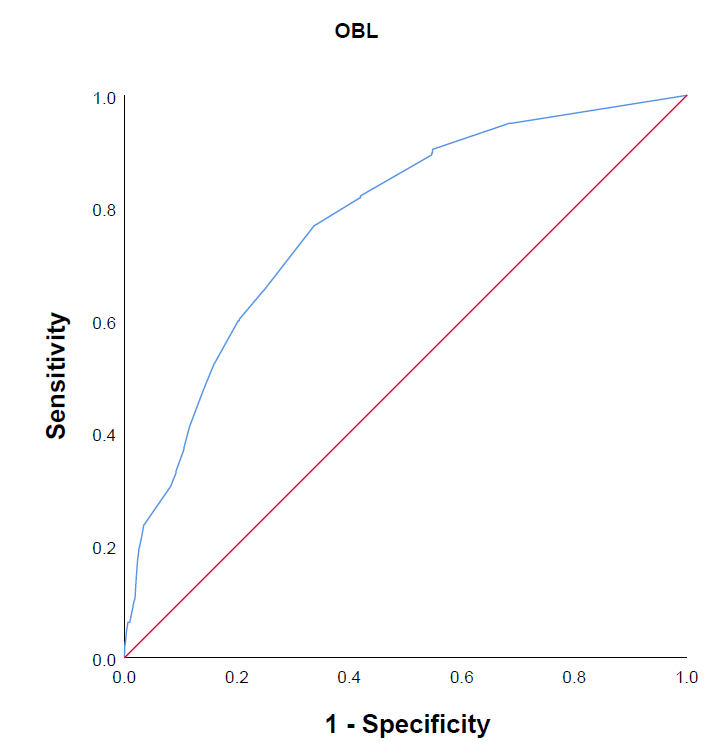
Figure S1. Receiver Operating Characteristic (ROC) curve among adolescents with astigmatism ≥2 dioptres, stratified by astigmatism axis groups.**
The ROC curve evaluates the diagnostic performance for keratoconus detection across different astigmatism axis groups: with-the-rule (WTR), against-the-rule (ATR), and oblique (OBL). The corresponding AUC values are shown for each group.

AUC = 0.744, 95% CI: 0.729–0.758, p < 0.001

AUC = 0.775, 95% CI: 0.750–0.801, p < 0.001

AUC = 0.775, 95% CI: 0.748–0.802, p < 0.001
